# Dental stem cell sphere formation and potential for neural regeneration: A scoping review

**DOI:** 10.1016/j.heliyon.2024.e40262

**Published:** 2024-11-08

**Authors:** Mohammed S. Basabrain, Ahmed Zaeneldin, Mohammed Nadeem Bijle, Chengfei Zhang

**Affiliations:** a Restorative Dental Sciences, Faculty of Dentistry, Umm Al-Qura University, Makkah, Saudi Arabia; b Restorative Dental Sciences, Faculty of Dentistry, The University of Hong Kong, Hong Kong, SAR, China; c Paediatric Dentistry, Faculty of Dentistry, The University of Hong Kong, Hong Kong SAR, China

**Keywords:** Adult stem cells, Central nervous system, Dental and periodontal, 3-D cell culture

## Abstract

**Background:**

Dental stem cells with neurosphere-forming abilities are a promising cell source for the treatment of neural diseases and injuries. This scoping review aimed to systematically map the existing literature on dental sphere formation assays and their characteristics associated with neural regeneration potential.

**Methods:**

The Web of Science, EMBASE, SCOPUS, and PubMed databases were systematically searched for *in vitro*, animal, and clinical studies and reviews focusing on stem cells isolated from the oral cavity, subsequently cultured as spheres with neural regeneration potential. Data were extracted and evidence was synthesized according to the predetermined variables in the registered protocol.

**Results:**

A total of 35 articles (31 *in vitro*, 1 combined *in vitro* and *in vivo*, and 3 reviews) were included. The predominant method utilized for sphere formation was low-attachment culture. Spheres were characterized using assessment of neural marker expression *via* confocal microscopy, immunohistochemistry, RT-qPCR, or western blotting. Overall, the synthesized results indicate a lack of *in vivo* studies investigating the utility of dental neurospheres for neural regeneration, with dental pulp stem cells being the most investigated for their neural regenerative potential.

**Conclusion:**

Dental stem cell spheres demonstrate significant potential for neural regeneration. Several assays and characterizations have been performed to characterized the mechanisms underlying dental sphere formation. Furthermore, *in vivo* studies are imperative to deduce the neural regenerative potential of stem cells in complex biological environments.

## 1 Introduction

Neurological illnesses and injuries can significantly affect a patient's quality of life, leading to neural impairments such as hypoesthesia, paralysis, or dementia [1]. Several approaches, such as axotomy, providing a suitable environment, and inhibiting specific proteins, have been employed to enhance neural regeneration [2,3,4]. However, the success rate of such treatments is low [1], making it imperative to explore interventions that aid in effectuating neural regeneration with limited risks and higher success rates.

Replicating clinical conditions to identify suitable treatments is crucial [5]. Through decades of investigation, researchers have realized that cells behave differently when cultured in three-dimensional environments. 3D culture methods provide a more complex environment through cell-cell interactions, the extracellular matrix, and different adhesin complexes than 2D models [6]. As such, most previous studies have overlooked the fact that two-dimensional cell culturing does not fully represent the three-dimensional environment in organisms [5]. As such, developing a model for stem cell utilization is necessary to close the gap between *in vitro* and clinical conditions.

Neural regeneration using stem cell therapy is a promising approach. Embryonic stem cells appear favorable for neural regeneration, but are subject to extensive ethical debate [7]. Therefore, identifying alternatives is important. In addition, obtaining neural stem cells (NSCs) from adults was thought to be impossible until reports on neurosphere formation developed through the colonization of dissociated cells from the central nervous system to form floating spheres [8]. NSCs are expected to possess three fundamental prerequisites: clonal expansion, multilineage differentiation, and self-renewal [9]. Based on this assumption, NSCs are considered the gold standard for neural regeneration [10].

Following the initial isolation of NSCs from adults via sphere formation, several studies have investigated the beneficial effects of sphere formation [11,12,13]. The results showed that sphere formation can better mimic *in vivo* cell growth, promote self-renewal, prevent cell differentiation, and maintain the cells’ original properties [11,12,13]. However, surgical procedures are required to isolate NSCs, commonly leading to donor-site morbidity. As such, the application of human NSCs is restricted by their limited sources and low expansion capacity [14]. Therefore, it is vital to explore cell lines with neural regenerative potential that would otherwise not raise concerns about ethics, isolation sources, and extraction measures.

Mesenchymal stem cells (MSCs) are prevalent across various tissues because of their crucial role in tissue maintenance and repair [15]. Various *in vitro* studies have reported that MSCs derived from bone marrow and adipose tissue can potentially differentiate into neural or Schwann cell-like cells [8,1]. However, due to senescence induction and donor age, adipose-derived stem cells may not be able to differentiate into Schwann cell-like cells for a long time [16]. Bone marrow stem cells have been extensively studied; however, retaining their eventual fate can be challenging [17,18]. Although stem cell therapies have been investigated in preclinical and clinical studies [3,7], advancements have been limited, thus highlighting the need to explore novel therapies while extending their potential for clinical applications.

Dental stem cells originating from the neural crest during embryonic development have mesenchymal and neural potential owing to their ectomesenchymal origin. Under specific conditions, cells express neural markers, differentiate into neurogenic lineages, and release neurotrophic factors under specific conditions [19,20]. Moreover, they have demonstrated efficacy in treating transacted spinal cords by reducing inflammation, promoting regeneration, and preventing hemorrhagic necrosis [21]. However, long-term 2D culture and serial passages may induce changes in stem cells, resulting in the loss of self-renewal abilities and culminating in senescence [16]. Therefore, 3D culture is promising for neural regeneration and can overcome the shortcomings of dental stem cells.

Dental stem cells include apical papilla stem cells (SCAPs), dental pulp stem cells (DPSCs), periodontal ligament stem cells (PLDSCs), stem cells from human exfoliated deciduous teeth (SHEDs), and dental follicle stem cells (DFSCs). Multiple techniques for generating dental spheres have been attempted (e.g., coated, non-coated, and low attachment plates; different media; and static or dynamic culture environments) [22,23,24,25]. Furthermore, some studies have tested their self-renewal ability through secondary sphere formation [26,27]. Indeed, several assays have been conducted to characterize the effects of dental spheres on neural cell stemness, including those utilizing RT-qPCR, Western blot, immunofluorescence, etc. [27,28,29]. However, existing methods for aerial comprehension have not yet been summarized in an orderly manner. As such, this scoping review aimed to systematically map the literature on dental sphere formation assays and their characteristics associated with neural regeneration potential.

## 2 Methods

The current scoping review was conducted in accordance with a seminal paper on scoping reviews [30]. The information in this paper was reported as per the PRISMA extension for scoping reviews (PRISMA-ScR) checklist for obligatory reporting items [31]. The research topic was conceptualized using the SPICE framework in accordance with the focus of the review.

S – Setting/study type: Any

P – Perspective: Dental sphere formation for neural regeneration

I – Intervention: Sphere formation

C – Comparison: Single layer culturing

E − Evaluation: Formation assays and associated characterizations

### 2.1 Protocol registration

Before initiating the review, all reviewers collectively defined, evaluated, and refined the study protocols. The finalized registered protocol included a list of reviewers, review objectives, search strategy with duration, study inclusion/exclusion criteria, data charting variables, and synthesis process. The pilot-tested registered protocol for public access will be available at https://osf.io/m73gc from August 2023. It should be noted that this protocol was strictly adhered to throughout the review.

### 2.2 Record sources, study search, selection, and inclusion

#### 2.2.1 Record sources

Literature databases including the Web of Science, EMBASE, SCOPUS, and PubMed were searched for studies published up to August 2023, using an optimized search strategy developed after identifying keywords pertinent to the research topic and applying appropriate Boolean operators. A supplemental reference search of qualifying papers was undertaken and internal reviewers (ZC and MNB) were consulted to gain professional insights. Only records available in the databases as titles and abstracts in English were considered for further examination. Duplicates were removed from the final search outcomes using EndNote 21 (Clarivate, Philadelphia, USA) to facilitate the progression to study selection. Two independent reviewers (MB and AZ) performed the search according to a predetermined literature search protocol.

#### 2.2.2 Study eligibility

Peer-reviewed records pertaining to dental neurosphere formation for neural regeneration were scanned based on the titles and abstracts to determine eligibility for inclusion in the review. The inclusion criteria were as follows: *in vitro*, animal, clinical studies, and reviews investigating stem cells isolated from the oral cavity, and subsequently cultured as spheres with the potential for neural regeneration. The exclusion criteria were: studies that lacked relevant keywords, or did not specifically address the review question, research protocols, commentaries/opinions, or papers published in languages other than English were excluded. To ensure a comprehensive coverage of the topic, additional searches were conducted on the reference lists of the included articles.

#### 2.2.3 Search strategy

The search strategy was based on the fundamental elements of the designed SPICE framework, as described in the protocol. The identified keywords with Boolean operators were as follows: (“apical papilla” OR “pulp” OR “periodontal ligament” OR “dental follicle” OR “dental epithelial” OR “cervical loop” OR “gingival” OR “dental stem cell∗") AND (“sphere” OR “dental sphere” OR “dental sphere formation”) AND (“neural” OR “regeneration” OR “potential” OR “neural regeneration” OR “neural potential”). The final comprehensive search string for PubMed is presented in Table S. Neither limitations nor filters were applied to the search strategy before the relevant keyword entries in the search fields of the respective databases.

#### 2.2.4 Study inclusion

After removing duplicates, two reviewers (MB and AZ) evaluated the identified records for consistency. Only studies that met the eligibility criteria were included. Two reviewers (MB and AZ) sequentially and independently assessed the title, abstract, and full text of each article. Any disagreements were resolved by discussion with a third reviewer (ZC or MNB).

### 2.3 Data variables and extraction

The designed protocol served as a template to extract variables such as article characteristics (study type, author (year)), dental sphere formation methods (primary sphere formation, secondary sphere formation, and duration), characterization methods for marker expression (confocal microscopy, immunohistochemistry, RT-qPCR, and western blotting), results (significant data), and neurosphere-related conclusions. As per the registered protocol, the data-charting process was performed using a piloted data-charting sheet *ab initio*. Two reviewers (MB and AZ) independently executed this process using MS Excel 2023 for Mac version 16.77 (Microsoft, Richmond, USA). Any discrepancies during the charting procedure were resolved by consultation with a third reviewer (ZC or MNB).

### 2.4 Synthesis of evidence

Following data abstraction, the research papers were summarized according to dental stem cell types and sources. Articles focusing on sphere formation assays and characterization identified during primary data extraction were synthesized at this review stage. As the review process did not include any evaluation of the evidence, a critical assessment of the resources was not undertaken.

## 3 Results

### 3.1 Study selection process

**Fig. 1:**
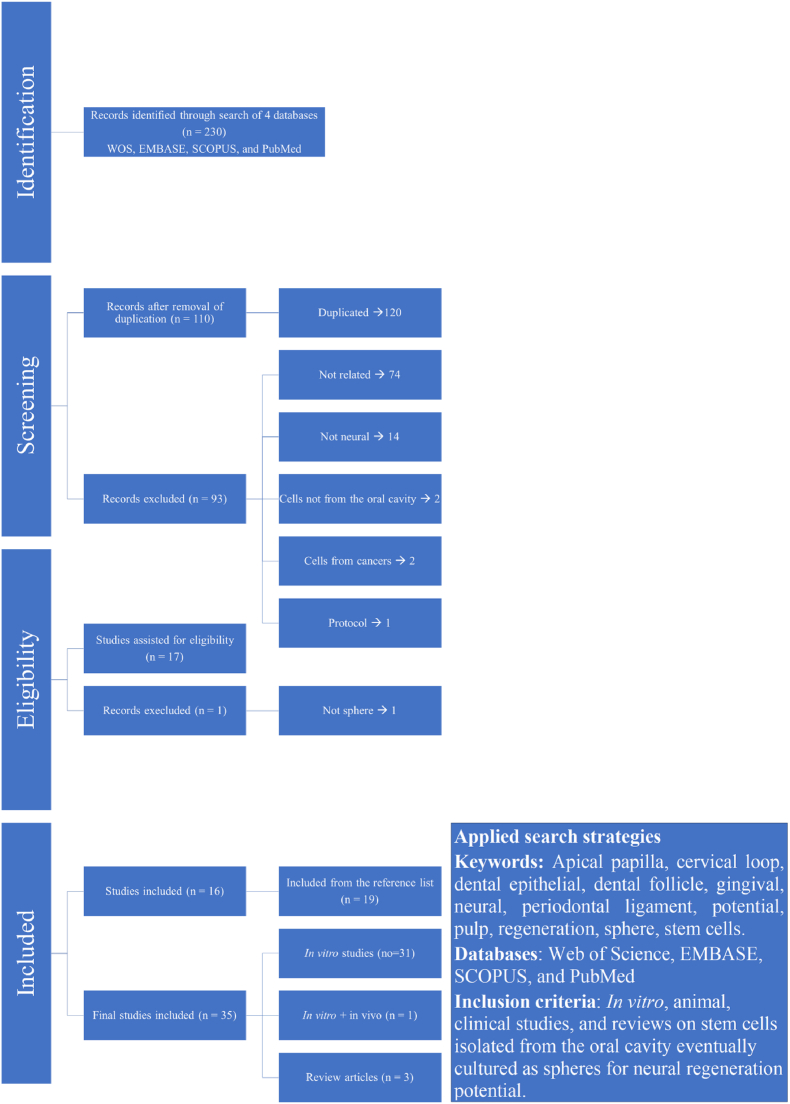
Flow diagram of study search, selection, and inclusion.

The study selection process for the present review is presented in Fig. 1. In summary, 230 records were initially identified from predetermined databases. From these, 120 were duplicates and 94 were excluded for the reasons outlined in the flowchart in Fig. 1. Subsequently, 17 publications were assessed for eligibility for inclusion in the systematic review. An additional 19 articles were identified and included from a review of the reference lists after examining the titles and abstracts. When the inclusion process was deemed complete, the level of agreement between all reviewers was 100 %.

### 3.2 Characteristics of the included studies

Most of the included articles were *in vitro* studies [[22], [26], [32], [33],^34^,^35^,^23^,[36], [37], [38],^39^,[40], [41], [42],^24^,^43^,^44^,^45^,^46^,^47^,^27^,^28^,^48^,^49^,[25], [29], [50], [51], [52],^53^,^54^], but we also included three reviews [55,56,57] and one combined *in vitro* and *in vivo* study [58]. The *in vitro* studies included were categorized based on cell type and origin. Seven types of dental stem cells were identified in the included articles: SCAPs, DPSCs, PDLSCs, DFSCs, SHEDs, gingival stem cells (GSCs), and oral mucosa stromal stem cells (OMSCs). Data extracted from the *in vitro* studies are presented in Table 1.

**Table 1:** A summary of relevant *in vitro* & *in vivo* studies.

No.	Author (Year)	Stem cell source	Dental sphere formation method Sphere formation method	Media	Characterization			Results	Conclusion
					Induction/formation	Marker expression	Self-renewal capacity		
					Experimental				
					Duration				
***In vitro*** **SCAPs isolated from humans**									

1	Abe, Hamada [22] (2011)	Human/SCAPs	Low attachment	Serum-free DMEM/F12 + bFGF, EGF	Duration: 7 days	Test used: IF – Nestin, MAP-2, Musashi-1, β-tubulin III	NA	Nestin, Musashi-1 (+) β-tubulin III, MAP-2 (−)	Stem/progenitor cells derived from SCAP spheres express markers of neural progenitor stem cells.
2	Abe, Hamada [32] (2012)	Human/SCAPs	Low attachment	Serum-free DMEM/F12 + bFGF, EGF, N2 Neural cell differentiation: 1- High-glucose DMEM + FBS 2- High-glucose DMEM + FBS, NGF, bFGF, dbcAMP, IBMX, RA Spheres seeding: Poly L-ornithine coating	Formation: 7 days Induction: 1–7 days 2–7 days	Test used: RT-PCR – Nestin, Musashi-1, Snaill, Slug, p75, β-tubulin III, MAP-2	Secondary spheres (✓)	Undifferentiated spheres: Nestin, Musashi-1, Snaill, Slug, p75 (+) β-tubulin III, MAP-2 (−) Differentiated spheres: Nestin, Musashi-1 (−) β-tubulin III, MAP-2 (+)	SCAP spheres are a reliable source of cells for regenerating neural crest lineage tissues.
3	Li, Xiang [24] (2020)	Human/SCAPs	Medium	Sphere formation: DMEM/F12 + EGF, FGF2, N-2, B-27, P/S Digested spheres seeding: Matrigel coating	Formation: 7 days	Test used: IF --------------p75NTR, HNK-1	NA	p75NTR, HNK-1 (+)	At the right concentration, PRP can encourage NCSCs, derived from SCAPs, to proliferate, remain viable, and differentiate into odontogenic cells.
4	Basabrain, Zhong [34] (2020)	Human/SCAPs	Low attachment (microtissue mold)	⍺-MEM + FBS, P/S Spheres seeding: Rat tail collagen-I coating	Formation: 3 days Duration: 4 & 7 days	Test used: ELIZA, IF, RT-PCR – Oct-4, Sox-2, Sox-10, Nestin, β-tubulin III, MAP-2, NeuN, NSE, NFM, BDNF, NGF	NA	SCAPs spheres: Oct-4, Sox-2, Nestin, β-tubulin III, MAP-2, NeuN, NSE, NFM, BDNF, NGF (increased) Sox-10 (no difference)	The formation of SCAP spheres enhances their neurogenic potential.

***In vitro*** **DPSCs isolated from humans**									

5	Stevens, Zuliani [49] (2008)	Human/DPSCs	Medium	Sphere formation: MesoCult MSC basal medium + bFGF, EGF, B27	Formation: 4–7 days	Test used: IHC – Nestin, CD271	Secondary spheres (✓) up to 5 passages	Nestin, CD271 (+)	DPSCs demonstrated label-retaining and sphere-forming capacities, which are attributed to multipotent neural crest stem cells.
6	Karbanová, Soukup [42] (2011)	Human/DPSCs	Medium	Neurogenic expansion: neurogenic + BMP-2 Sphere formation: Neurobasal + B27, bFGF, EGF, BMP-2, L-glutamine, P/S	Expansion 2D: 3 weeks Formation: 7 days	Test used: IF, IHC – Nestin, A2B5, β-tubulin III, O4	NA	Nestin, A2B5, β-tubulin III, O4 (+)	Generated neurospheres showed peripheral expression of both immature and mature neural markers, indicating the potential for differentiation into several types of neural cells.
7	Xiao and Tsutsui [54] (2013)	Human/DPSCs	Medium	Sphere formation: MEM‐a + knockout serum replacement Proliferation: MEM‐a + FBS, GlutaMAX, ascorbic acid 2‐phosphate	Formation: 24 h Induction: 1, 4, 15 weeks Proliferation: 7 days	Test used: IF, RT-qPCR – P75, HuC/D, CDH2, CD24, NFM, β-tubulin III	NA	1 week: P75 (+) central HuC/D (+) intermediate 15 weeks: CDH2 (increased) 1, 4, and 15 weeks spheroids vs. control monolayer: CD24, NFM, β-tubulin III (increased)	The spheroid model utilized serves as a valuable research tool to explore the molecular basis of stem cell homeostasis and tissue organization, holding substantial potential for neural tissue regeneration. For long-term survival of stem cells and differentiation of DPSC spheres into neural tissue, maintaining membrane integrity, the presence of fibroblast growth factor, and cell adhesion are imperative.
8	Osathanon, Sawangmake [45] (2014)	Human/DPSCs	Medium	Sphere formation: Neurobasal medium + FGF, EGF, B27, L-glutamine, amphotericin B, P/S Dissociated sphere seeding: Collagen-IV coating Neural cell differentiation: Same medium + RA	Formation: 7 days Induction: 7 days	Test used: IF, RT-PCR – β-tubulin III, Sox2, Sox9	NA	β-tubulin III, Sox2, Sox9 (increased)	An approach involving the induction of growth factors is the preferred protocol to facilitate the differentiation of DPSCs into neurons.
9	Gervois, Struys [38] (2015)	Human/DPSCs	Low attachment	DMEM/F12 + B27, EGF, bFGF, P/S Neural cell differentiation: Neurobasal medium + l-glutamine, B27, N2, dbcAMP, NT-3, P/S Sphere seeding: poly-l-ornithine (PLO) Laminin coating	Formation: 6–8 days Induction: 4 weeks	Test used: ELIZA, IF, RT-PCR, Transmission electron microscopy – Neun, MAP2, NCAM, GAP43, Synapsin I, Synaptophysin, GFAP, Nestin, BDNF, VEGF, NGF, GDNF	NA	Neun, MAP2, NCAM, GAP43, Synapsin I, Synaptophysin, GFAP (+) Nestin, BDNF (decrease) VEGF, NGF (increase) GDNF (maintained)	The findings of this study demonstrate that DPSCs can differentiate into neurons during neurosphere formation, displaying various morphological and electrophysiological characteristics of functioning neuronal cells.
10	Chun, Soker [23] (2016)	Human/DPSCs	Gelatin-coated plates stage 1 poly-L-ornithine/fibronectin-coated stage 5	5 stages: Human/Mouse Dopaminergic Neuron Differentiation Kit 1- Culturing: KO-ES medium + LIF for 3–4 days. 2- Sphere formation: KO-ES medium without LIF 3- Selection of Nestin (+) cells from spheres: ITS medium + fibronectin 4- Cells expansion: N-2 medium + bFGF, FGF-8b, sonic hedgehog-N, ascorbic acid 5- Neural cell differentiation: N-2/ascorbic acid medium	Culturing: 4 days Formation: 4 days Selection: 6–8 days Expansion: 2 weeks Induction: 10–15 days	Test used: IF, RT-PCR – SSEA4, Nestin, β-tubulin III, GFAP, MBP, TH, Vimentin, Pax6, O4	NA	SSEA4 (+) stages 1–4/(−) stage 5 TH (+) in stage 5 only Nestin, β-tubulin III, O4 (variable expression) GFAP, Vimentin (+) MBP, Pax6 (increased)	DPSCs demonstrated the capability to differentiate into dopaminergic neurons, showcasing their potential as an autologous cell source for the treatment of Parkinson's disease (PD).
11	Fatima, Khan [36] (2017)	Human/DPSCs	Medium	Sphere formation: Serum-free human neural proliferation medium + EGF, bFGF, antibiotic solution Neural cell differentiation: Human neural differentiation medium + RA, FBS, without EGF, bFGF Spheres formation in: - Adherent - Non-adherent	Formation: 3 up to 21 days Induction: 21 days	Test used: IF, Molecular characterization, RT-qPCR – Nestin, NCAM, NF-M, PLP1, O4, BDNF, GDNF, NGF, GFAP, MAP2, β-tubulin III	NA	Nestin, NCAM, NF-M, PLP, O4, BDNF, GDNF, NGF, GFAP, MAP2, β-tubulin III	Treated DPSCs formed either attached or floating spheres. Floating spheres have higher proliferation abilities and contain more than one stem cell population, including neural precursor stem cells.
12	Pisciotta, Bertoni [46] (2018)	Human/DPSCs	Low attachment	Serum-free DMEM/F12 + B27, EGF, b-FGF, L-glutamine, P/S Neural cell differentiation: Serum-free DMEM/F12 + B27, EGF, b-FGF, N2, NGF, BDNF L-glutamine, all-trans-RA, P/S Spheres seeding: Poly-L-lysine	Formation: 24 h Induction: 3 weeks	Test used: IF, Western blot – Nestin, CD271, Sox-10	Secondary spheres (✓) up to 8 passages	Before differentiation: Nestin, CD271, Sox-10 (+) After differentiation: MAP-2 (+) Nestin (decreased) β-tubulin III (increased)	Neural crest-derived DPSCs can preserve their biological characteristics in a favorable microenvironment *via* 3D sphere culture.
13	Niapour, Hamidabadi [43] (2019)	Human/DPSCs	Medium	Sphere formation: DMEM/F12 + bFGF, EGF, B27, L-glutamine, NEAA, P/S Neural cell differentiation: Neurobasal medium + B27, N2, dbcAMP, NT-3, L-glutamine, P/S Treatment: Notch signalling inhibitor DAPT Spheres seeding: poly-lysine coating	Formation: 7 days Induction: 3 weeks	Test used: RT-qPCR, RT-PCR, IF – MAP2, Nestin, Pax-6, hes1, hey1, Sox-1, Ascl1, Neurogenin2	NA	Upon Notch signalling inhibitor: Nestin, Pax-6, Sox-1, hes1, hey1 (decreased) Ascl1, Neurogenin2, MAP-2 (increased)	Notch signalling activity is key in stemness and neurosphere generation.
14	Rafiee, Pourteymourfard-Tabrizi [47] (2020)	Human/DPSCs	Low attachment	Knockout DMEM/KO DMEM/F12 + P/S, with/without EGF, bFGF heparin Spheres seeding: Fibronectin or poly-lysine coating	Formation: 24 h Duration: 3 & 10 days	Test used: IF, RT-qPCR, Western blot – Nestin, Sox2, MAP2, Neurogenin 1 (Ngn1)	NA	EGF, bFGF, heparin: Nestin, Sox2, MAP2, Ngn1 (increase)	EGF, bFGF, and heparin enhance the differentiation of DPSC spheres towards neural and progenitor cells.
15	Solis-Castro, Boissonade [48] (2020)	Human/DPSCs	Low attachment	DFNB: P/S, bFGF, EGF Treatments: 1- Standard medium: DMEM, Glutamax, Ascorbic Acid, FBS 2- OSCFM: DFNB DMEM:F12, N2, B27, bFGF, IGF, 20 EGF, P/S 3- BMP4 medium: OSCFM supplemented with BMP4 Neural cell differentiation: DFNB, NT3, dbcAMP Spheres seeding: Polyornithine/Laminin coating	Formation: 6–8 days Induction: 2 weeks	Test used: IF, RT-qPCR – Sox2, Sox9, Nestin, SLUG, SNAIL1, AP2a, Sox10, P75, HNK1, Peripherin, β-tubulin III, Tau	NA	Spheres: Sox2 (+) Spheres and monolayer: Sox9, Nestin, SLUG, SNAIL1 (+) Spheres vs. monolayer: P75, SNAIL1 (increased) Sox10, AP2a (increased only in a few samples) BMP4 spheres: AP2a, Sox10, P75, HNK1, SNAIL1 (increased) Neural differentiation: Peripherin, β-tubulin III (increased) Tau (only in BMP4 group)	The DPSC sphere enhances neural potentials, while BMP4 further enhances both stemness and neural differentiation.

***In vitro*** **DPSCs/Animals**									

16	Takeyasu, Nozaki [52] (2006)	Rat/DPSCs	Low attachment	Neural cell differentiation: D-MEM/F-12 + glucose, L-glutamine, NaHCO, N-2 Plus Media + bovine insulin, human transferrin, putrescine, selenite, progesterone, EGF, FGF Cells seeding: Poly-lysine coating	Induction: 2 weeks	Test used: IF, RT-qPCR – Nestin, β-tubulin III, MAP2, GFAP	NA	After induction: Nestinl MAP-2 (decreased) GFAP (increased) β-tubulin III (maintained)	Neural stem cells can be sourced from external neural entities, with DPSCs being one example.
17	Sasaki, Aoki [27] (2008)	Rat/DPSCs	Low attachment	serum-free culture medium DMEM ⁄ F12 + B27, P/S - EGF and bFGF were added after 4–5 days Spheres seeding: poly-l-ornithine and fibronectin coating	Formation: 7–9 days After seeding: 7–14 days	Test used: IF – β-tubulin III, MAP2, S100	Secondary spheres (✓)	β-tubulin III, MAP2, S100 (+)	Cells that are double-positive for CD81 and Nestin, found in the odontoblast layer of the dental pulp's apical region, may have the capability to develop into neurospheres. The formation of neurospheres from DPSCs is enhanced by the addition of bFGF.

***In vitro*** **PDLSCs isolated from humans**									

18	Huang, Pelaez [40] (2009)	Human/PDLSCs	Low attachment	DMEM + FBS, β-mercapto-ethanol, sodium pyruvate, nonessential amino acids, glutamine, B27, retinoid acid, P/S Spheres seeding: Gelatin-coating	Formation: 4 days Induction: 7 days	Test used: IF, RT-PCR – MAP2, GFAP, NFM, β-tubulin III	NA	MAP2, GFAP, NFM, β-tubulin III (+)	PDL may harbor neural crest-derived pluripotent stem cells.
19	Osathanon, Manokawinchoke [44] (2013)	Human/PDLSCs	Medium	Spheres formation: Neurobasal medium + bFGF, EGF, B27, l-glutamine, amphotericin B, P/S Neural cell differentiation: The neurogenic medium + RA Sphere seeding: Collagen IV coating Or Surface-bound Notch ligand, Jagged-1	Formation: 1–7 days Induction: 7 days	Test used: IF, RT-PCR, RT-qPCR – β-tubulin III, NF, Sox2, Sox9, Hes1, Hey1	NA	In floating spheres vs. monolayer: β-tubulin III, NF, Sox2, Sox9, Hes1, Hey1 (increased) In attached vs. floating spheres: β-tubulin III, NF (increased) Sox2, Sox9, Hes1, Hey1 (decreased) Surface-bound Jagged-1: β-tubulin III, NF, Hes1, Hey1 (increased)	The Notch signaling pathway potentially plays a role in the formation and maturation of neurospheres.
20	Sawangmake, Pavasant [28] (2014)	Human/PDLSCs	Medium	Spheres formation: Neurobasal medium + bFGF, EGF, B27, l-glutamine, amphotericin B, P/S Neural cell differentiation: The neurogenic medium + RA Treatment with or without: - Dglucose - Mannose Sphere seeding: Collagen IV coating	Formation: 1–7 days Induction: 7 days	Test used: IF, RT-PCR, RT-qPCR – β-tubulin III, NF, NM, Sox2, Sox9, Hes1, Hey1	NA	β-tubulin III, NF, NM, Sox2, Sox9, Hes1, Hey1 (increased) Glucose during formation: Sox2, Hes1, Hey1 (decreased) Glucose during maturation: All markers (no difference)	High glucose environments adversely impact neurosphere formation.

***In vitro*** **PDLSCs isolated from animals**									

21	Techawattanawisal, Nakahama [29] (2007)	Rat/PDLSCs	Low attachment	Serum-free basal medium + EGF, bFGF, LIF Neural cell differentiation: Basal medium + FBS, BDNF, NGF-b, NT-3 Glial cell differentiation: Basal medium + FBS, human recombinant heregulin β, forskolin Spheres seeding: poly-L-lysine/laminin-coating	Formation: 2 days Induction: 2–3 weeks Glial induction: 1–2 weeks	Test used: IF, RT-PCR – GFAP, Nestin, NFM, CNPase, Twist, Slug, Sox2, Sox9	NA	Neural markers: GFAP, Nestin (+) Transcription factors: Twist, Slug, Sox2, Sox9 (+) Neural differentiation: NFM (+) Glacial differentiation: GFAP, CNPase (+)	Spheres formed by PDLSCs contain both neural and mesenchymal precursor stem cells, and favor neural differentiation.

***In vitro*** **DFSCs isolated from humans**									

22	Völlner, Ernst [53] (2009)	Human/DFCSs	Gelatin laminin poly-L-ornithine poly-L-lysine.	Neural differentiation: NSCM I: Neurobasal - medium containing L-glutamine, G5-supplement, and neural stem cell supplement NSCM II Neurobasal - 1-medium containing L-glutamine, B27, EGF, FGF-2, 2-medium containing L-glutamine, B27, RA For NSCM III Neurobasal - medium containing B27 and RA	Induction NSCM-I: 7 days Induction NSCM-II: 4 + 7 days Duration: 11 days Induction NSCM-III: 7 days	Test used: IF, RT-qPCR – Nestin, β-tubulin III, NSE, Neurofilament, GAL, Tachykinin	NA	Neurosphere like cluster: Nestin, β-tubulin III, NSE, Neurofilament, GAL, Tachykinin (+)	The two-step process for neuronal differentiation results in the differentiation of DFCs into neural-like cells.
23	Kanao, Ogura [41] (2017)	Human/DFCSs	Low attachment	DMEM + B27, EGF, FGF Neural cell differentiation: MSC Neurogenic differentiation medium Spheres seeding: Fibronectin coating	Formation: 2 days Induction: 7 days	Test used: IF, RT-qPCR – Musashi-1, Musashi-2, MAP2, GFAP, MBP, Sox10, Nestin, β-tubulin III	NA	Spheres vs. monolayer: Nestin, Sox10, MAP2, MBP (increase) Spheres induced vs. non-induced: Musashi-1, Musashi-2, MAP2, GFAP, MBP, Sox10 (increase) Nestin, β-tubulin III (decrease)	For cell-based therapies targeting neurological illnesses, hDFCs may represent an ideal additional source of neural/glial cells.

***In vitro*** **DFSCs isolated from animals**									

24	Beck, Petersen [35] (2011)	Mouse/DFCSs	Poly-L-Lysine coating	Serum-replacement medium (SRM) DMEM/F-12 + N2, FGF-2, EGF, P/S,	Formation: Cells were cultured until non-adherent spherical clusters were formed within 7 days	Test used: Proteomics	NA	73 % of 676 identified similar proteins in DFSCs and retinal Muller cells, and 55 % of them are expressed in the brain	Spheres derived from different cell types exhibit highly similar proteomes. Which likely reflect essential cellular processes occurring in neurosphere-like cell clusters.

***In vitro*** **SHEDs isolated from humans**									

25	Wang, Wang [58] (2010)	Human/SHEDs	Low attachment	Neurobasal A media + B27, bFGF, EGF Neural cell differentiation: SHH, FGF8, GDNF, forskolin Sphere seeding: poly- l -lysine-coated	Duration: 7–9 days Induction: 7 days	Test used: IF, RT-qPCR, Western blot – Nestin, β-tubulin-III, TH, MAP2	NA	Nestin, β-tubulin-III (+) TH (−) After induction: β-tubulin-III, Nestin (decreased) MAP2, TH (increased)	SHEDs may represent an optimal source of cells for Parkinson's disease therapy.
26	Su and Pan [50] (2016)	Human/SHEDs	Low attachment (PDMS)	RSC DMEM + FBS Media movements: 2- Static 2- Dynamic Sphere seeding: glass coverslip	Duration: 3, 7, & 11 days Static formation: 7 days Dynamic formation: 3–7 days Sphere seeding: 11 days	Test used: IF, RT-qPCR – Nestin, β-tubulin-III, GFAP, γ-enolase	NA	After 7 days: Nestin, β-tubulin-III (+) After 11 days: GFAP, γ-enolase (+) After seeding: Nestin, β-tubulin-III (decreased) GFAP, γ-enolase (increased)	These findings suggest a new approach for treating nerve damage through the differentiation of SHEDs by using RSC culture media.
27	Su, Shih [25] (2016)	Human/SHEDs	Porous chitosan	αMEM + FBS L-ascorbic acid 2-phosphate, bFGF Media movements: 2- Static 2- Dynamic Neural cell differentiation: Neurobasal A media containing B27, EGF, bFGF	Formation: 2 days Induction: Static/dynamic: 5 & 10 days	Test used: IF, RT-qPCR – GFAP, Nestin, γ-enolase, β-III-tubulin, CNPase, BCL2/BAX	NA	In dynamic compared to static: GFAP, Nestin, γ-enolase, β-III-tubulin, CNPase, BCL2/BAX (increased)	Culturing SHEDs in porous chitosan under dynamic conditions leads to neurosphere formation. The formed neurospheres are capable of glial cell differentiation.
28	Su, Pan [51] (2018)	Human/SHEDs	Low attachment (PDMS)	RSC DMEM high glucose + FBS Sphere seeding: glass coverslip	Formation: 7 days Duration: 7, 15 days	Test used: IF, RT-qPCR – Nestin, β-tubulin-III, GFAP, γ-enolase	NA	Sphere: Nestin, β-tubulin-III, GFAP, γ-enolase (increased) After seeding: Nestin, β-tubulin-III (decreased) GFAP, γ-enolase (increased)	Neurospheres from SHEDs can be more effectively induced in a low-attachment PDMS, and can differentiate into glial cells using an RSC medium.

***In vitro*** **GSCs isolated from humans**									

29	Fournier, Loison-Robert [37] (2016)	Human/GSCs	Low attachment	NS4 = DMEM/F12 + EGF, FGF CM = DMEM + FBS Neural cell differentiation: NeuM1 = DMEM, FBS, RA in CM for 10 days NeuM2 = DMEM/F12, N2, FGF2 for 3 weeks Sphere seeding: Poly-L-Lysine coating	Formation: 5 days Duration: 7 days	Test used: IF, RT-qPCR – Snai1, Twist1, Pax3, Sox9, Nestin, FOXD3, Tau, Type I collagen, Tenascin-C, S100B, β-tubulin-III, MAP2, NFM, GFAP	NA	Attached sphere: Nestin, tenascin-C, Sox9 (increased) Type I collagen (decreased) Floating spheres: Sox9, Nestin, Tenascin-C, S100B, MAP2 (increased) Pax3, FOXD3, Snai1, Twist1 (maintained) Nestin, β-tubulin-III (decreased) MeuM1induced attached spheres: Tau (increased) NeuM2-induced attached spheres: NFM, GFAP (increased)	Benefiting from floating sphere cultures may allow human GSC cultures to serve as a reliable source of NC-derived cells.
***In vitro*** **OMSCs isolated from humans**									
30	Abe, Yamaguchi [26] (2016)	Human/OMSCs	Low attachment	Serum-free DMEM/F12 + N2, bFGF, EGF Neural cell differentiation: Neurogenic-promoting medium Abe et al. (2012)	Formation: 7 days Induction: 7 days	Test used: IF, Microarray, RT-qPCR, RT-PCR – Nestin, CD44, Slug, Snail, MSX1, Hes1, Sox9, β-tubulin-III, NeuroD1, NSE, GFAP	Secondary spheres (✓)	Nestin, CD44, Slug, Snail, MSX1, Hes1, Sox9 (+) After induction: β-tubulin-III, NeuroD1, NSE, GFAP (+)	Spheres exhibit populations enriched for neural characteristics, possessing self-renewal and multipotency capabilities.

***In vitro*** **Stem cells from different origins isolated from humans**									

31	Gonmanee, Thonabulsombat [39] (2018)	Human/DPSCs &SHEDs	Low attachment	DMEM/F12, + bFGF, EGF, B-27, P/S Disassociated sphere seeding: poly-L-ornithine, laminin Neural cell differentiation: DMEM/F12 + BDNF, NT-3, GDNF, N-2, B-27, P/S	Formation: 5 days Induction: 2 weeks	Test used: IF, RT-qPCR – β-tubulin-III, GFAP, TrkB, GATA3, NTRK2	NA	β-tubulin-III, TrkB, GATA3, NTRK2 (+) GFAP (rarely detected)	Cells derived from DPSC and SHED spheres exhibited comparable abilities to differentiate into functional SGN neurons, influenced by BDNF, NT-3, and GDNF.
32	Abe, Kaida [33] (2022)	Human/SCAPSs, PDLSC s & OMSCs	Low attachment	Serum-free DMEM/F12 + bFGF, EGF, N2 Neural cell differentiation: MSC neurogenic differentiation medium	Formation: 7 days	Test used: IF, Microarray, RT-qPCR, RT-PCR – NES, CD44, SNAI1, SNAI2, MSX1, Hes1, Nestin	NA	All spheres with varied expression of NES, CD44, SNAI1, SNAI2, MSX1, Hes1, Nestin Neural differentiation: All spheres expressed β-tubulin-III	SCAPs, PDLSCs, and OMSCs exhibit sphere-forming abilities with variable expression of neural and progenitor markers, dependent on tissue origin, which should be considered prior to use.

***In vivo*** **SHEDs isolated from humans**									

1	Wang, Wang [58] (2010)	Human/SHEDs	Low attachment	Neurobasal Medium Sphere formation: Neurobasal A media + bFGF, EGF	Formation: 7 days In-Rat: 2, 4, 6, and 8 weeks	TH	NA	Medium: TH (−) SHEDs & SHED spheres: TH (+)	SHED spheres hold significant potential for the treatment of Parkinson's disease.

Achaete-Scute Homolog 1 (ASCL1)/Brain-Derived Neurotrophic Factor (BDNF)/Basic Fibroblast Growth Factor (bFGF)/Bone Morphogenetic Protein (BMP)/Cyclic Adenosine Monophosphate (cAMP)/N-[N-(3,5-Difluorophenacetyl- L-Alanyl)]-Sphenylglycine T-Butyl Ester (DAPT)/Dibutyryl Camp (dbcAMP)/Dental Pulp Stem Cell (DPSC)/Dental Follicle Cells (DFSCs)/Dulbecco's Modified Eagle's Medium (DMEM)/Epidermal Growth Factor (EGF)/Fetal Bovine Serum (FBS)/Growth Associated Protein 43 (GAP43)/Glial Cell-Derived Neurotrophic Factor (GDNF)/Glial Fibrillary Acidic Protein (GFAP)/Gingival Stem Cells (GSCs)/Human Natural Killer-1 (HNK-1)/Neural Hu Proteins (Huc/D)/3-Isobuthyl-1-Methyl Xanthine (IBMX)/Immunofluorescence (IF)/Insulin-Transferrin-Sodium (ITS)/Knockout-Embryonic Stem Cell (KO-ES)/Leukaemia Inhibitory Factor (LIF)/Microtubule-Associated Protein 2 (MAP2)/Myelin Basic Protein (MBP)/Minimum Essential Medium (MEM)/Mesenchymal Stem Cell (MSC)/Neural Cell Adhesion Molecule (NCAM)/Neural Crest Stem Cells (NCSCs)/N-Cadherin (CDH2)/Non-Essential Amino Acid (NEAA)/Neurofilament Medium Chain (NFM)/Nerve Growth Factor (NGF)/Neuron-Specific Enolase (NSE)/Oligodendrocytic (O4)/Octamer-Binding Transcription Factor 4 (OCT4)/Oral Mucosa Stromal Stem Cells (OMSCs)/Otic Stem Cell Full Medium (OSCFM)/P75 Neurotrophin Receptor (P75ntr)/Paired Box 6 (PAX6)/Periodontal Ligament Stem Cells (PDLSCs)/Polydimethylsiloxane (PDMS)/Platelet-Rich Plasma (PRP)/Penicillin, Streptomycin (P/S)/Retinoic Acid (RA)/Rat Schwann Cell (RSC)/Stem Cells From Apical Papilla (SCAP)/Stem Cells From Human Exfoliated Deciduous Teeth (SHED)/Stage-Specific Embryonic Antigen-4 (SSEA-4)/Tyrosine Hydroxylase (TH)/Vascular Endothelial Growth Factor (VEGF).

### 3.3 Investigations

#### 3.3.1 *In vitro* studies

Several *in vitro* studies were conducted to demonstrate the potential of dental and oral stem cells in neural regeneration, as outlined in Table 1. Assays for sphere formation included studies on primary sphere formation, secondary sphere formation, and the formation mechanism. Characterization assays encompassed the expression of various progenitor, mature, immature, and apoptotic markers, and investigations of neural differentiation, cell morphology, and neural cell function (Table 1).

##### 3.3.1.1 Dental sphere formation assays

The basic principles of neurosphere generation lie in the culturing methods which could be more beneficial for neural differentiation [38]. Spheres can be generated on low-attachment, coated, and non-treated culture surfaces using fresh culture media. The low attachment method was the most prevalent, applied in 19 studies [[22], [26], [32], [33],^34^,^37^,^38^,^39^,^40^,^41^,^46^,^47^,^27^,^48^,^50^,^51^,^52^,^29^,^58^]. Of these, two studies employed polydimethylsiloxane (PDMS) as a low attachment scaffold [50,51], while two studies used agarose as a coating or microtissue mold for sphere generation owing to its poor attachment properties, [34,40]. The coating method is the least used for sphere generation [35,23,53]. For non-treated culturing surfaces, nine studies used serum-free culture media for sphere formation [36,42,24,43,44,45,28,49,54], with neurobasal medium being the most prevalent [42,44,45,14]. The culturing media were typically supplemented with EGF, bFGF, and B27 [42,43,44,45,14,49]. Using this method without any surface treatment, two types of spheres were generated: adherent and non-adherent. Non-adherent spheres formed faster and exhibited superior proliferation ability compared with adherent spheres [36,54]. Floating spheres also showed increased expression of progenitor and immature neural markers, whereas attached spheres induced the neural cell maturation [37,44].

Secondary sphere formation is an indicator of self-renewal capacity [26,27], and was shown to be superior in human dental stem cells to that of rat stem cells, indicating higher stemness in cells isolated from humans [32,26,46,27,49].

Sphere formation assays were conducted using a variety of observation methods including microscopic examination [27,28,29], histological sectioning [54], and electron microscopy [38].

##### 3.3.1.2 Characterizations

Diverse characterization techniques, such as PCR, western blotting, immunofluorescence, microarray, and protein isolation, were employed throughout the different studies, as listed in Table 1. Cells were characterized to identify cell types, maturity, and differentiation towards neural stem cells. All of the included studies used one or more of the following neural markers: Nestin, β-tubulin-III, and MAP2 [[22], [26], [32], [33],^34^,^23^,[36], [37], [38],^39^,[40], [41], [42],^24^,^43^,^44^,^45^,^46^,^47^,^27^,^28^,^48^,^49^,[25], [29], [50], [51], [52],^53^,^58^,^54^], except for that by Beck & Petersen [35], which focused on assessing neural proteomics rather than gene expression.

The identification of cell types within spheres or induced spheres is necessary for their technical applications in the management of neurodegenerative diseases. For example, Chun & Soker [23] showed that the cells could differentiate into dopaminergic neurons by expressing Nestin, β-tubulin III, GFAP, MBP and TH. Similarly another study showed that dopaminergic neurons could be obtained by inducing the formation of dental spheres from MAP-2, β-tubulin III, and TH-positive cells [58]. Spheres could differentiate into glial cells neural lineage by down-regulating Nestin and upregulating glial fibrillary acidic protein (GFAP) [52]. Dental sphere cells could further be induced to differentiate into ganglion neuron-like cells through induction with BDNF, NT-3, and GDNF [39]. Furthermore, dental stem cells can also differentiate into neuron-like cells following the expression of multiple neural and progenitor markers, such as Nestin, Sox10, MAP2, and MBP [41]. Eventually, differentiated dopaminergic neurons can be used as an autologous cell source for patients with PD [23,58]. As such, dental sphere formation and differentiation towards specific neural lineages can be used in neural regeneration.

Neurosphere culturing methods possess several advantages over 2D culturing methods. Sphere formation positively influences cell stemness, which can be further improved through the optimization of culturing methods. In addition, sphere formation increases neural stemness by upregulating CD44, which explains apoptosis resistance [46,49]. Sphere treatment with platelet-rich plasma (PRP) increased p75NTR and HNK-1 expression, promoted cell proliferation, and maintained cell viability [24]. The expression of progenitor, immature, and neural markers (Sox2, Sox9, and β-tubulin III, respectively) was duly boosted by adding growth factors [45]. Heparin-treated neurospheres had neural stem cell markers (Nestin, Sox2, MAP2 and Ngn1) that were upregulated shortly after induction, and within ten days, the cells exhibited a neural-like morphology [47]. Treatment of spheres with BMP4 further enhanced neural crest differentiation and phenotype [48]. The growth factors listed in the included articles were studied for neural regeneration and differentiation (Table 1) to explain their effects on sphere formation.

All of the included studies showed the neurogenerative potential of dental neurospheres *via* neural marker expression; however, only four articles conducted electrophysiological measurements to confirm normal neural function [33,38,39,46]. Two studies used intracellular calcium imaging, with the results indicating normal neural activities [33,39]. The other two recorded the neural activity of the cells *via* patch clamps on the migrating cells out of the spheres, and the results revealed the ability of the cells to fire an action potential [38,46].

#### 3.3.2 The *in vivo* study

In the single included *in vivo* study, Wang et al. [58] used a rat model to explore the neural regeneration potential of SHEDs for PD treatment. Three groups were established: basic medium, intact SHEDs, and SHED. The experiment was extended for 8 weeks, during which the behavior of the rats was meticulously recorded. After two weeks, the SHED spheres demonstrated significant behavioral improvement compared to the monolayer. As such, the use of SHED spheres for the treatment of Parkinson's appears to be highly promising.

#### 3.3.3 Reviews

We identified three pertinent reviews on this subject, as listed in Table 2 [55,56,57]. Of these, Heng and Lim [55] reviewed dental and oral neural induction protocols, Luo et al. [57] emphasized the role of DPSCs in neural regeneration, and Ko and Chen [56] offered a succinct review of SHEDs. The utilization of dental stem cells in neural regeneration is a recurring theme in these reviews, and aligns well with our objectives. Consequently, we explored additional relevant articles from their reference lists, as indicated in Table 2, which are summarized in Table 1.

**Table 2:** Summary of the relevant literature reviews.

SN	Author (Year)	Articles included from reference lists
1	Heng, Lim [55]	Review/DPSC, PDLSC, SHED and DFSC Studies (9) 1 Völlner, Ernst [53] 2 Huang, Pelaez [40] 3 Wang, Wang [58] 4 Karbanová, Soukup [42] 5 Osathanon, Manokawinchoke [44] 6 Xiao and Tsutsui [54] 7 Osathanon, Sawangmake [45] 8 Sawangmake, Pavasant [28] 9 Gervois, Struys [38]
2	Luo, He [57]	Review/DPSCs Studies (8) 1 Karbanová, Soukup [42] 2 Xiao and Tsutsui [54] 3 Osathanon, Sawangmake [45] 4 Gervois, Struys [38] 5 -Chun, Soker [23] 6 Heng, Lim [55] 7 Fatima, Khan [36] 8 Gonmanee, Thonabulsombat [39]
3	Ko, Chen [56]	Review/SHED Studies (4) 1 Wang, Wang [58] 2 Su, Shih [25] 3 Su and Pan [50] 4 Su, Pan [51]

#### 3.3.4 Synthesis of results

This scoping review encompassed 31 *in vitro* studies [[22], [26], [32], [33],^34^,^23^,[36], [37], [38],^39^,[40], [41], [42],^24^,^43^,^44^,^45^,^46^,^47^,^27^,^28^,^48^,^49^,[25], [29], [50], [51], [52],^53^,^54^], one combined *in vitro* and *in vivo* study [58], and three reviews [55,56,57], as detailed in Table 3. However, no clinical data were included in this review. Various dental stem cells including SCAPs, DPSCs, PDLSCs, DFSCs, SHEDs, GSCs, and OMSCs generate neurospheres. DPSCs have been the most extensively studied [[22], [32], [33],^34^,^24^], whereas OMSCs [33,26] and GSCs [37] have been the least studied for neural regeneration (Table 1, Table 3). Regardless of the methods employed for sphere formation, whether through low attachment, regular plates, coated plates, or sphere-forming culture medium (Table 1), all studies successfully generated spheres. Further, all studies highlighted the substantial potential of dental sphere formation for neural regeneration (Table 3).

**Table 3:** Data synthesis.

SN	Stem cells source	Study types and references	Synthesized interpretation	Neural regeneration potential
1	SCAPs	• *In vitro*: [[22], [32], [33],^34^,^24^]. •Review: [55]	Five *in vitro* studies and one review article utilized human SCAP spheres for neural regeneration.	All studies confirmed the neural regeneration potential of human SCAPs, with none demonstrating any limitations or adverse effects associated with this potential.
2	DPSCs	•*In vitro*: [23,36,38,39,42,43,45,46,47,27,48,49,52,54] •Reviews: [55,57]	Fourteen included studies on DPSCs were *in vitro,* while two were review articles. Twelve of these studies utilized DPSCs from humans, while two employed DPSCs from rats.	All studies corroborated the neural regeneration potential of human DPSCs, with none displaying any limitations or adverse effects associated with this potential.
3	PDLSC	•*In vitro*: [33,40,44,28,29] •Review: [55]	Five of the included studies on PDLSCs were *in vitro*, and one was a review. Of these, four utilized stem cells from humans and one from rats.	All studies affirmed the neural regeneration potential of human PDLSCs, with none revealing any limitations or adverse effects associated with this potential.
4	DFSCs	•*In vitro*: [35,41,53]	Three studies on DFSCs were *in vitro*; two utilized human stem cells, and one employed mouse stem cells.	All studies endorsed the use of DFSC spheres for neural regeneration.
5	SHEDs	•*In vitro* + *In vivo*: [58] •*In vitro*: [39,[25], [50], [51]] •Review: [56]	SHEDs were the only stem cells we could identify in our comparison of *in vitro* and *in vivo* studies. Of these, six were *in vitro* studies, and one was a review article. All *in vitro* studies employed human stem cells.	All included articles supported the use of SHED spheres for neural regeneration.
6	GSCs	•*In vitro*: [37]	Only one included study was *in vitro*, utilizing human GSCs.	This study supported the use of GSC spheres for neural regeneration.
7	OMSCs	•*In vitro*: [33,26]	Two *in vitro* studies with human OMSCs were included.	Both articles supported the use of OMSC spheres for neural regeneration.

## 4 Discussion

The discovery of sphere formation represents a significant breakthrough in neural stem cell biology [8]. Sphere generation enables the retrospective identification of multipotent neural stem cells capable of differentiating into neurons, astrocytes, and oligodendrocytes [59]. It also serves as a reliable method for identifying NSCs within neurogenic sites in the adult brain [60]. Dental stem cells have demonstrated significant neural potential because of their origin in the neural crest. Under suitable conditions, these cells can express neural markers, differentiate into neurogenic lineages, and release neurotrophic factors [19,20]. Understanding the mechanisms underlying dental sphere formation at both the molecular and functional levels could facilitate a better understanding of the etiology of neurodegenerative diseases. Dental stem cells have the potential to address prevailing challenges related to accessibility, plasticity, and ethical compatibility. Thus far, several studies have employed dental stem cells in treating different neurodegenerative diseases (e.g. spinal cord injury, Alzheimer's disease, Parkinson's disease, and strokes) [23,61,58]. SCAPs, DPSCs, and DFSCs have been used to treat transacted spinal cords, reduce inflammation, promote regeneration, and prevent hemorrhagic necrosis [21].

This review systematically maps the literature on dental stem cell sphere formation assays and their associated characteristics to investigate neural regeneration potential. To date, multiple methods have been employed to induce neurosphere formation, including environmental modifications of the culture medium, surface alteration, and culture dynamics.

Starting with sphere formation through the culture medium, several different types of media were employed with or without serum, with different growth factors and other additives [23,36,43,44,28]. Spheres formed in serum-containing media without supplements demonstrate elevated levels of progenitor, immature, and mature neuronal markers compared to monolayers [34]. Similarly, when SCAPs were cultured as spheres in serum-free media, an elevation in neural markers was observed [[22], [32], [33],^24^]. Sasaki et al. [27] further explored the capability of DPSCs to form neurospheres, discovering that sphere formation relied on exogenous bFGF, but not EGF. Supplementation of bFGF in the culture medium enhanced the number and size of dental sphere formation, whereas factors such as TGF-β and glucose exerted a negative effect [43,27,28,62]. Fournier et al. [37] compared various culture media and assessed them based on the number and size of spheres generated. These findings indicated that the most efficacious sphere-forming medium incorporated EGF, FGF, and B27. Concurrently, treating the spheres with platelet-rich plasma (PRP) elevated the expression of p75NTR and HNK-1, increased cellular proliferation, and preserved viability [24]. Nevertheless, the most commonly used medium was serum-free DMEM/F12 [32,22,26,38,39,24,43,46,47,27,48,52,58], followed by neurobasal medium [33,38,42,44,45,28,49,53,58] (Table 1). Thus, selection of the culture medium is crucial for optimal neural sphere formation and regeneration.

Sphere formation on different surfaces can significantly influence neurosphere formation; as such, various modifications have been explored (Table 1). Surfaces such as plastic glass, coated surfaces, and low attachment plates are examples of the different surfaces used [34,35,40,49,51,25,53]. Herein, we divide the surfaces into untreated, attached, and low-attachment surfaces, discussing each individually. In one study, Fatima et al. [36] cultured cells on untreated plates for sphere formation between days 1 and 21. In this study, two types of spheres were formed: adherent and floating. The results showed that floating spheres showed superior cell proliferation. In contrast, spheres formed within 4–7 days on plates coated with laminin, poly-L-ornithine, poly-L-lysine, and gelatin [53]. Conversely, the utilization of low-attachment plates allows sphere formation within 1–7 d, which is emerging as the preferred method. Several low-attachment surfaces, such as agarose and porous chitosan, were used in these studies [34,25]. Agarose microtissue molds offer advantages in controlling sphere size and number [34]. Further studies are required to compare the effects of different surface modifications on sphere formation.

Finally, sphere formation using dynamic or static culturing methods was tested; however, only two studies were identified [50,25]. In this method, medium flow is regulated by a peristaltic pump and is set at 0.8 ml/min. The effects of dynamic and static culture environments on SHEDs have been previously examined and compared [50,25]. In a dynamic culture environment, cells begin to aggregate into spheres after three days, and the number of spheres increases over time [50]. Furthermore, the Bcl-2/Bax ratio was measured following the application of an apoptotic stimulus, revealing that dynamic culturing enhances cell survival capability [25].

Secondary sphere formation is indicative of self-renewal capability [32,49]. Of the 32 studies investigated, only five involved secondary sphere-forming assays, with two conducting secondary sphere-formation assays only once [32,26,46,27,49]. The results of these studies showed that both the number and size of the secondary spheres were inferior to those of the primary spheres [32,26]. As highlighted by Sasaki et al. [27], rat DSPCs can generate secondary spheres once. In contrast, human DPSCs have demonstrated the ability to produce secondary spheres for up to 8 passages [46,49]. Given the indicative nature of their self-renewal potential, secondary sphere formation assays should be prioritized when selecting stem cells for neural regeneration.

Several neural characterization assays were conducted in the literature to demonstrate the neural regeneration potential of the dental spheres. The spheres were tested to identify the cell types within them (Table 1). For example, proteomic measurements from different cell type spheres exhibit approximately 55 % resemblance to neural cells [35]. Moreover, the maturity levels of the cells within the spheres could be determined through marker expression. Numerous studies have illustrated that sphere formation leads to the upregulation of various progenitor, mature, and immature neural markers [34,48,54]. Characterization of the sphere of dental stem cells will be discussed based on modification of the culture medium, expression of neural markers, and its potential for use in treating neurodegenerative diseases.

Modification of the culture media could lead to neural differentiation of the spheres into specific neural cell types. For example, treating neurospheres with heparin results in the upregulation of neural stem cell markers (Nestin, Sox2, MAP2, and Ngn1); shortly after induction, these cells manifest a neural-like morphology within ten days [47]. In addition, treating spheres with BMP4 further enhances neural crest differentiation and phenotype [48]. The use of growth factors leads to the amplification of progenitors and the expression of immature neural markers such as Sox2, Sox9, and β-tubulin III [45]. Finally, BDNF, NT-3, and GDNF can induce the differentiation of dental sphere cells into ganglion neuron-like cells [39].

The maturity and differentiation of DSCs can be evaluated through the expression of neural markers such as Nestin, Sox10, MAP2, and MBP [41]. In some studies, marker expression was tested to compare the dental stem cell spheres before and after induction. The majority of the included studies presented a consistent trend of upregulation of mature neural markers (MAP2, tyrosine hydroxylase (TH), GFAP, and MPB) and downregulation of immature markers (nestin) [32,46,58]. However, the expression of Musashi-1 and β-tubulin-III exhibited significant variability post-differentiation [41,45,46,52,58], which could be attributed to the use of different stem cells or culture environments.

Dental sphere formation and differentiation toward specific neural lineages could be used to manage neurodegenerative diseases, such as Parkinson's disease and stroke. Several studies showed the ability of dental spheres to differentiate into dopaminergic neurons by expressing Nestin, β-tubulin III, MAP-2, GFAP, MBP, and TH. Dopaminergic neurons have the potential to serve as autologous cell sources in patients with Parkinson's disease [23,58]. Thus, transplantation of dopaminergic neurons from SHED spheres was used for the treatment of Parkinsonian rats and showed improvement within 2 weeks [58]. Takeyasu and Nozaki [52] indicated the potential of spheres to differentiate into the glial cell neural lineage, marked by the downregulation of nestin and upregulation of GFAP. Dental stem cells have demonstrated significant potential *in vivo* for stroke treatment by minimizing the infarct volume and increasing the migration and differentiation of endogenous neural progenitor cells [61]. As such, dental stem cells are potential candidates for the treatment of neurodegenerative diseases.

The cellular properties of dental spheres have been extensively investigated [38,46,54]. Within the spheres, slow-proliferating central mother cells retain longer labels than fast-proliferating peripheral daughter cells [46,54]. Furthermore, sphere formation leads to the upregulation of neurotrophins, such as BDNF, GDNF, and NGF, along with their receptors, including P75 [34,36,48]. Sphere formation results in the upregulation of CD44, and enhances resistance to apoptosis [46,49]. The maintenance of cell membrane integrity, cell adhesion, and fibroblast growth factor expression are some of the outcomes of sphere formation [54]. A detailed examination of the DPSC spheres revealed enhanced metabolic activity, intracellular communication, and cell signaling. The results indicated an increase in vesicular transport, highlighting the role of intracellular communication via paracrine signaling. Additionally, higher expression of cAMP is important for maintaining neural differentiation, while elevated NT-3 signaling is crucial for neurogenic maturation [38]. Neurosphere formation enhances the expression of Notch signaling target genes Hes1 and Hes2. Additionally, the use of the Notch inhibitor (γ-secretase inhibitor, DAPT) led to the formation of smaller spheres and had a significant effect on both target genes. This underscores the critical role of the Notch signaling pathway in preserving neural stemness and facilitating sphere formation [44]. These findings suggest that prolonged proliferative ability, differentiation, self-renewal, and apoptosis resistance enhance dental stem cell neurosphere formation [38,46,49,54].

The primary aim of this review was to collect and summarize available information from all sources on dental sphere formation assays and their associated characterizations. Hence, a critical appraisal of the included articles was not conducted [63,31]. Another limitation of this review is the exclusion of non-English literature from our search because all contributors comprehended the language in common.

### 4.1 Gaps and future scopes of research

This review elucidates the advantageous effects of sphere formation by dental stem cells on neural regeneration through improved abilities in terms of proliferation, apoptosis resistance, membrane integrity maintenance, cell adhesion preservation, and prolonged cell survival when subjected to sphere formation (Table 1, Table 3). However, further characterization assays are required to ensure a more refined understanding of the signaling pathways involved.

The stemness of sphere cells can be further enhanced by optimizing culturing methods using treatments such as PRP, growth factors, heparin, and BMP4. These approaches promote cell proliferation, maintain cell viability, and improve the expression of neural markers and differentiation. Exploring these methods further could be a promising area for future research [24,45,47,48].

Further electrophysiological studies are required to explore neural physiological functions. For example, the organ-on-chip model provides a sophisticated environment for assessing cellular interactions in the treatment of neurodegenerative diseases. Furthermore, our scoping review highlights a deficiency of *in vivo* studies concerning dental sphere formation in neural regeneration. Therefore, researchers exploring this subject should consider employing *in vitro* data in a preclinical milieu to identify the potential for *in vivo* experimental outcomes.

## 5 Conclusion

Based on the evidence identified in this review, the following conclusions can be drawn:

1. Dental stem cell spheres demonstrate immense potential in neural regeneration.
2. Several assays and associated characterizations have been utilized in the past, with the low-attachment method being frequently employed for sphere formation. Nestin, β-tubulin-III, and MAP2 are the predominant neural markers used to characterize neural regenerative potential.
3. Among the different stem cells with neural regenerative capabilities explored for dental sphere formation, DPSCs are the most commonly used.

Further *in vivo* studies on this subject are imperative, given the significant potential of dental spheres in neural regeneration.

## CRediT authorship contribution statement

**Mohammed S. Basabrain:** Writing – review & editing, Writing – original draft, Methodology, Investigation, Formal analysis, Data curation, Conceptualization. **Ahmed Zaeneldin:** Writing – review & editing, Methodology, Investigation, Data curation. **Mohammed Nadeem Bijle:** Writing – review & editing, Supervision, Methodology, Formal analysis. **Chengfei Zhang:** Writing – review & editing, Supervision, Project administration, Methodology, Funding acquisition, Conceptualization.

## Impact statement

This review highlights the use of neurosphere culture methods to enhance the neurological potential of dental stem cells. However, there is a notable lack of *in vivo* studies employing dental spheres to treat neurodegenerative diseases.

## Data availability statement

The datasets used and/or analyzed in the present study are available from the corresponding author upon reasonable request.

## Ethics declaration

Review and/or approval by an ethics committee was not required because this study was a scoping review.

## Declaration of competing interest

The authors declare the following financial interests/personal relationships which may be considered as potential competing interests:CF ZHANG reports financial support was provided by The National Natural Science Foundation of China (81970934, 82170938) and the RGC General Research Fund, Hong Kong (Ref No. 17111619, 17115420, 17124821). If there are other authors, they declare that they have no known competing financial interests or personal relationships that could have appeared to influence the work reported in this paper.

## References

[1] Wang D., Wang Y., Tian W., Pan J. (2019). Advances of tooth‐derived stem cells in neural diseases treatments and nerve tissue regeneration. Cell Prolif..

[2] Laabs T., Carulli D., Geller H.M., Fawcett J.W. (2005). Chondroitin sulfate proteoglycans in neural development and regeneration. Curr. Opin. Neurobiol..

[3] F So K., Aguayo A. (1985). Lengthy regrowth of cut axons from ganglion cells after peripheral nerve transplantation into the retina of adult rats. Brain Res..

[4] Zörner B., Schwab M.E. (2010). Anti‐Nogo on the go: from animal models to a clinical trial. Ann. N. Y. Acad. Sci..

[5] Pampaloni F., Reynaud E.G., Stelzer E.H. (2007). The third dimension bridges the gap between cell culture and live tissue. Nat. Rev. Mol. Cell Biol..

[6] Bissell M.J., Rizki A., Mian I.S. (2003). Tissue architecture: the ultimate regulator of breast epithelial function. Curr. Opin. Cell Biol..

[7] Thomson J.A., Itskovitz-Eldor J., Shapiro S.S., Waknitz M.A., Swiergiel J.J., Marshall V.S., Jones J.M. (1998). Embryonic stem cell lines derived from human blastocysts. Science.

[8] Reynolds B.A., Weiss S. (1992). Generation of neurons and astrocytes from isolated cells of the adult mammalian central nervous system. Science.

[9] Marshall G.P., Reynolds B.A., Laywell E.D. (2007). Using the neurosphere assay to quantify neural stem cells in vivo. Curr. Pharmaceut. Biotechnol..

[10] Steward M.M., Sridhar A., Meyer J.S., Ellen Heber-Katz D.L.S. (2013). New Perspectives in Regeneration.

[11] Gil‐Perotín S., Duran‐Moreno M., Cebrián‐Silla A., Ramírez M., García‐Belda P., García‐Verdugo J.M. (2013). Adult neural stem cells from the subventricular zone: a review of the neurosphere assay. Anat. Rec..

[12] Pastrana E., Silva-Vargas V., Doetsch F. (2011). Eyes wide open: a critical review of sphere-formation as an assay for stem cells. Cell Stem Cell.

[13] Recabal A., Caprile T., García-Robles M.d.l.A. (2017). Hypothalamic neurogenesis as an adaptive metabolic mechanism. Front. Neurosci..

[14] Siemionow M., Brzezicki G. (2009). Current techniques and concepts in peripheral nerve repair. Int. Rev. Neurobiol..

[15] Brand-Saberi B. (2020).

[16] Faroni A., Smith R.J., Lu L., Reid A.J. (2016). Human Schwann‐like cells derived from adipose‐derived mesenchymal stem cells rapidly de‐differentiate in the absence of stimulating medium. Eur. J. Neurosci..

[17] Cuevas P., Carceller F., Dujovny M., Garcia-Gómez I., Cuevas B.A., González-Corrochano R., Diaz-González D., Reimers D. (2002). Peripheral nerve regeneration by bone marrow stromal cells. Neurol. Res..

[18] Cuevas P., Carceller F., Garcia-Gómez I., Yan M., Dujovny M. (2004). Bone marrow stromal cell implantation for peripheral nerve repair. Neurol. Res..

[19] Kim B.-C., Bae H., Kwon I.-K., Lee E.-J., Park J.-H., Khademhosseini A., Hwang Y.-S. (2012). Osteoblastic/cementoblastic and neural differentiation of dental stem cells and their applications to tissue engineering and regenerative medicine. Tissue Eng., Part B Rev..

[20] Parisi L., Manfredi E. (2016). Applicability of tooth derived stem cells in neural regeneration. Neural Regen. Res..

[21] Yang C., Li X., Sun L., Guo W., Tian W. (2017). Potential of human dental stem cells in repairing the complete transection of rat spinal cord. J. Neural. Eng..

[22] Abe S., Hamada K., Yamaguchi S., Amagasa T., Miura M. (2011). Characterization of the radioresponse of human apical papilla-derived cells. Stem Cell Res. Ther..

[23] Chun S.Y., Soker S., Jang Y.-J., Kwon T.G., Yoo E.S. (2016). Differentiation of human dental pulp stem cells into dopaminergic neuron-like cells in vitro. J. Kor. Med. Sci..

[24] Li J., Xiang L., Guan C., Yang X., Hu X., Zhang X., Zhang W. (2020). Effects of platelet-rich plasma on proliferation, viability, and odontogenic differentiation of neural crest stem-like cells derived from human dental apical papilla. BioMed Res. Int..

[25] Su W.T., Shih Y.A., Ko C.S. (2016). Effect of chitosan conduit under a dynamic culture on the proliferation and neural differentiation of human exfoliated deciduous teeth stem cells. J. Tissue Eng. Regen. Med..

[26] Abe S., Yamaguchi S., Sato Y., Harada K. (2016). Sphere‐derived multipotent progenitor cells obtained from human Oral mucosa are enriched in neural crest cells. Stem Cells Transl Med.

[27] Sasaki R., Aoki S., Yamato M., Uchiyama H., Wada K., Okano T., Ogiuchi H. (2008). Neurosphere generation from dental pulp of adult rat incisor. Eur. J. Neurosci..

[28] Sawangmake C., Pavasant P., Chansiripornchai P., Osathanon T. (2014). High glucose condition suppresses neurosphere formation by human periodontal ligament‐derived mesenchymal stem cells. J. Cell. Biochem..

[29] Techawattanawisal W., Nakahama K., Komaki M., Abe M., Morita Y. TakagiI. (2007). Isolation of multipotent stem cells from adult rat periodontal ligament by neurosphere-forming culture system. Biochem. Biophys. Res. Commun..

[30] Arksey H., O'Malley L. (2005). Scoping studies: towards a methodological framework. Int. J. Soc. Res. Methodol..

[31] Tricco A.C., Lillie E., Zarin W., O'Brien K.K., Colquhoun H., Levac D., Moher D., Peters M.D., Horsley T., Weeks L. (2018). PRISMA extension for scoping reviews (PRISMA-ScR): checklist and explanation. Ann. Intern. Med..

[32] Abe S., Hamada K., Miura M., Yamaguchi S. (2012). Neural crest stem cell property of apical pulp cells derived from human developing tooth. Cell Biol. Int..

[33] Abe S., Kaida A., Kanemaru K., Nakazato K., Yokomizo N., Kobayashi Y., Miura M., Miki T., Hidai C., Kitano H., Yoda T. (2022). Differences in the stemness characteristics and molecular markers of distinct human oral tissue neural crest-derived multilineage cells. Cell Prolif..

[34] Basabrain M.S., Zhong J., Luo H., Liu J., Yi B., Zaeneldin A., Koh J., Zou T., Zhang C. (2022). Formation of three-dimensional spheres enhances the neurogenic potential of stem cells from apical papilla. Bioengineering.

[35] Beck H.C., Petersen J., Felthaus O., Schmalz G., Morsczeck C. (2011). Comparison of neurosphere-like cell clusters derived from dental follicle precursor cells and retinal Müller cells. Neurochem. Res..

[36] Fatima N., Khan A.A., Vishwakarma S.K. (2017). Immunophenotypic and molecular analysis of human dental pulp stem cells potential for neurogenic differentiation. Contemp. Clin. Dent..

[37] Fournier B.P., Loison-Robert L.S., Ferré F.C., Owen G.R., Larjava H., Häkkinen L. (2016). Characterisation of human gingival neural crest-derived stem cells in monolayer and neurosphere cultures. Eur. Cell. Mater..

[38] Gervois P., Struys T., Hilkens P., Bronckaers A., Ratajczak J., Politis C., Brône B., Lambrichts I., Martens W. (2015). Neurogenic maturation of human dental pulp stem cells following neurosphere generation induces morphological and electrophysiological characteristics of functional neurons. Stem Cell. Dev..

[39] Gonmanee T., Thonabulsombat C., Vongsavan K., Sritanaudomchai H. (2018). Differentiation of stem cells from human deciduous and permanent teeth into spiral ganglion neuron-like cells. Arch. Oral Biol..

[40] Huang C.C., Pelaez D., Bendala J.D., Garcia-Godoy F., Cheung H.S. (2009). Plasticity of stem cells derived from adult periodontal ligament. Regen. Med..

[41] Kanao S., Ogura N., Takahashi K., Ito K., Suemitsu M., Kuyama K., Kondoh T. (2017). Capacity of human dental follicle cells to differentiate into neural cells in vitro. Stem Cell. Int..

[42] Karbanová J., Soukup T., Suchánek J., Pytlík R., Corbeil D., Mokrý J. (2011). Characterization of dental pulp stem cells from impacted third molars cultured in low serum-containing medium. Stem Cell. Int..

[43] Niapour A., Hamidabadi H.G., Niapour N., Mohammadi P., Pasandi M.S., Malekzadeh V. (2019). Pharmacological Notch pathway inhibition leads to cell cycle arrest and stimulates ascl1 and neurogenin2 genes expression in dental pulp stem cells-derived neurospheres. Biotechnol. Lett..

[44] Osathanon T., Manokawinchoke J., Nowwarote N., Aguilar P., Pavasant T. PalagaP. (2013). Notch signaling is involved in neurogenic commitment of human periodontal ligament-derived mesenchymal stem cells. Stem Cell. Dev..

[45] Osathanon T., Sawangmake C., Pavasant N. NowwaroteP. (2014). Neurogenic differentiation of human dental pulp stem cells using different induction protocols. Oral Dis..

[46] Pisciotta A., Bertoni L., Riccio M., Mapelli J., Bigiani A., La Noce M., Orciani M., de Pol A., Carnevale G. (2018). Use of a 3D floating sphere culture system to maintain the neural crest-related properties of human dental pulp stem cells. Front. Physiol..

[47] Rafiee F., Pourteymourfard-Tabrizi Z., Mahmoudian-Sani M.-R., Mehri-Ghahfarrokhi A., Soltani A., Hashemzadeh-Chaleshtori M., Jami M.-S. (2020). Differentiation of dental pulp stem cells into neuron-like cells. Int. J. Neurosci..

[48] Solis-Castro O.O., Boissonade F.M., Rivolta M.N. (2020). Establishment and neural differentiation of neural crest-derived stem cells from human dental pulp in serum-free conditions. Stem Cells Transl Med.

[49] Stevens A., Zuliani T., Olejnik C., Le H., Roy, Obriot H., Kerr-Conte J., Formstecher P., Bailliez Y., Polakowska R.R. (2008). Human dental pulp stem cells differentiate into neural crest-derived melanocytes and have label-retaining and sphere-forming abilities. Stem Cell. Dev..

[50] Su W.-T., Pan Y.-J. (2016). Stem cells from human exfoliated deciduous teeth differentiate toward neural cells in a medium dynamically cultured with Schwann cells in a series of polydimethylsiloxanes scaffolds. J. Neural. Eng..

[51] Su W.-T., Pan Y.-J., Huang T.-Y., Huang Y.-C. (2018). Hydrophobic PDMS promotes neural progenitor formation from SHEDs by Schwann cell–cultivated medium induction. Int. J. Polym. Mater. Polym. Biomater..

[52] Takeyasu M., Nozaki T., Daito M. (2006). Differentiation of dental pulp stem cells into a neural lineage. Pediatr. Dent. J..

[53] Völlner F., Ernst W., Driemel O., Morsczeck C. (2009). A two-step strategy for neuronal differentiation in vitro of human dental follicle cells. Differentiation.

[54] Xiao L., Tsutsui T. (2013). Characterization of human dental pulp cells‐derived spheroids in serum‐free medium: stem cells in the core. J. Cell. Biochem..

[55] Heng B.C., Lim L.W., Wu W., Zhang C. (2016). An overview of protocols for the neural induction of dental and oral stem cells in vitro. Tissue Eng. Part B Rev. Reviews..

[56] Ko C.-S., Chen J.-H., Su W.-T. (2020). Stem cells from human exfoliated deciduous teeth: a concise review. Curr. Stem Cell Res. Ther..

[57] Luo L., He Y., Wang X., Key B., Lee B.H., Li H., Ye Q. (2018). Potential roles of dental pulp stem cells in neural regeneration and repair. Stem Cell. Int..

[58] Wang J., Wang X., Sun Z., Wang X., Yang H., Shi S., Wang S. (2010). Stem cells from human-exfoliated deciduous teeth can differentiate into dopaminergic neuron-like cells. Stem Cell. Dev..

[59] Okano H. (2002). Neural stem cells: progression of basic research and perspective for clinical application. Keio J. Med..

[60] Gritti A., Bonfanti L., Doetsch F., Caille I., Alvarez-Buylla A., Lim D.A., Galli R., Verdugo J.M.G., Herrera D.G., Vescovi A.L. (2002). Multipotent neural stem cells reside into the rostral extension and olfactory bulb of adult rodents. J. Neurosci..

[61] Raza S.S., Wagner A.P., Hussain Y.S., Khan M.A. (2018). Mechanisms underlying dental-derived stem cell-mediated neurorestoration in neurodegenerative disorders. Stem Cell Res. Ther..

[62] Xiao L., Tsutsui T. (2013). Human dental mesenchymal stem cells and neural regeneration. Hum. Cell.

[63] Munn Z., Peters M.D., Stern C., Tufanaru C., Aromataris A. McArthurE. (2018). Systematic review or scoping review? Guidance for authors when choosing between a systematic or scoping review approach. BMC Med. Res. Methodol..

