# Supplementary material for: Dental stem cell sphere formation and potential for neural regeneration: A scoping review
Source: Heliyon. 2024 Nov 8;10(22):e40262. doi: 10.1016/j.heliyon.2024.e40262 (PMC11605411; doi:10.1016/j.heliyon.2024.e40262)
Supplement: Multimedia component 1 [file mmc1.docx]

**“Supplementary Table”**

**Table S:** Search strategy for PubMed.

| **Search #** | **Keyword** | **Records** | **Search details** |
| --- | --- | --- | --- |
| 1 | apical papilla | 1203 | ("apical"[All Fields] OR "apically"[All Fields] OR "apicals"[All Fields] OR "apices"[All Fields]) AND ("papilla"[All Fields] OR "papillae"[All Fields] OR "papillas"[All Fields]) |
| 2 | pulp | 56995 | "dental pulp"[MeSH Terms] OR ("dental"[All Fields] AND "pulp"[All Fields]) OR "dental pulp"[All Fields] OR "pulp"[All Fields] |
| 3 | periodontal ligament | 11794 | "periodontal ligament"[MeSH Terms] OR ("periodontal"[All Fields] AND "ligament"[All Fields]) OR "periodontal ligament"[All Fields] |
| 4 | dental follicle | 1843 | "dental sac"[MeSH Terms] OR ("dental"[All Fields] AND "sac"[All Fields]) OR "dental sac"[All Fields] OR ("dental"[All Fields] AND "follicle"[All Fields]) OR "dental follicle"[All Fields] |
| 5 | dental epithelial | 10411 | ("dental health services"[MeSH Terms] OR ("dental"[All Fields] AND "health"[All Fields] AND "services"[All Fields]) OR "dental health services"[All Fields] OR "dental"[All Fields] OR "dentally"[All Fields] OR "dentals"[All Fields]) AND ("epithelial"[All Fields] OR "epithelially"[All Fields] OR "epithelials"[All Fields]) |
| 6 | cervical loop | 4122 | ("cervic"[All Fields] OR "cervicals"[All Fields] OR "cervices"[All Fields] OR "neck"[MeSH Terms] OR "neck"[All Fields] OR "cervical"[All Fields] OR "uterine cervicitis"[MeSH Terms] OR ("uterine"[All Fields] AND "cervicitis"[All Fields]) OR "uterine cervicitis"[All Fields] OR "cervicitis"[All Fields]) AND "loop"[All Fields] |
| 7 | gingival | 65744 | "gingiva"[MeSH Terms] OR "gingiva"[All Fields] OR "gingival"[All Fields] OR "gingivally"[All Fields] OR "gingivals"[All Fields] OR "gingivitis"[MeSH Terms] OR "gingivitis"[All Fields] OR "gingivitides"[All Fields] |
| 8 | sphere | 56449 | "sphere"[All Fields] OR "sphere s"[All Fields] OR "spheres"[All Fields] |
| 9 | stem cell* | 555134 | ("plant stems"[MeSH Terms] OR ("plant"[All Fields] AND "stems"[All Fields]) OR "plant stems"[All Fields] OR "stem"[All Fields] OR "microscopy, electron, scanning transmission"[MeSH Terms] OR ("microscopy"[All Fields] AND "electron"[All Fields] AND "scanning"[All Fields] AND "transmission"[All Fields]) OR "scanning transmission electron microscopy"[All Fields]) AND "cell*"[All Fields] |
| 10 | dental sphere | 31 | (("dental health services"[MeSH Terms] OR ("dental"[All Fields] AND "health"[All Fields] AND "services"[All Fields]) OR "dental health services"[All Fields] OR "dental"[All Fields] OR "dentally"[All Fields] OR "dentals"[All Fields]) AND ("sphere"[All Fields] OR "sphere s"[All Fields] OR "spheres"[All Fields])) AND (review[Filter]) |
| 11 | dental sphere formation | 6 | (("dental health services"[MeSH Terms] OR ("dental"[All Fields] AND "health"[All Fields] AND "services"[All Fields]) OR "dental health services"[All Fields] OR "dental"[All Fields] OR "dentally"[All Fields] OR "dentals"[All Fields]) AND ("sphere"[All Fields] OR "sphere s"[All Fields] OR "spheres"[All Fields]) AND ("formations"[All Fields] OR "metabolism"[MeSH Terms] OR "metabolism"[All Fields] OR "formation"[All Fields])) AND (review[Filter]) |
| 12 | neural | 543049 | "neural"[All Fields] OR "neuralization"[All Fields] OR "neuralize"[All Fields] OR "neuralized"[All Fields] OR "neuralizes"[All Fields] OR "neuralizing"[All Fields] OR "neurally"[All Fields] |
| 13 | regeneration | 406472 | "regenerability"[All Fields] OR "regenerable"[All Fields] OR "regenerant"[All Fields] OR "regenerants"[All Fields] OR "regenerate"[All Fields] OR "regenerated"[All Fields] OR "regenerates"[All Fields] OR "regenerating"[All Fields] OR "regeneration"[MeSH Terms] OR "regeneration"[All Fields] OR "regenerations"[All Fields] |
| 14 | potential | 4154109 | "potential"[All Fields] OR "potential s"[All Fields] OR "potentialities"[All Fields] OR "potentiality"[All Fields] OR "potentially"[All Fields] OR "potentials"[All Fields] OR "potentiate"[All Fields] OR "potentiated"[All Fields] OR "potentiates"[All Fields] OR "potentiating"[All Fields] OR "potentiation"[All Fields] OR "potentiations"[All Fields] OR "potentiative"[All Fields] OR "potentiator"[All Fields] OR "potentiators"[All Fields] |
| 15 | neural regeneration | 46644 | "nerve regeneration"[MeSH Terms] OR ("nerve"[All Fields] AND "regeneration"[All Fields]) OR "nerve regeneration"[All Fields] OR ("neural"[All Fields] AND "regeneration"[All Fields]) OR "neural regeneration"[All Fields] |
| 16 | neural potential | 144202 | ("neural"[All Fields] OR "neuralization"[All Fields] OR "neuralize"[All Fields] OR "neuralized"[All Fields] OR "neuralizes"[All Fields] OR "neuralizing"[All Fields] OR "neurally"[All Fields]) AND ("potential"[All Fields] OR "potential s"[All Fields] OR "potentialities"[All Fields] OR "potentiality"[All Fields] OR "potentially"[All Fields] OR "potentials"[All Fields] OR "potentiate"[All Fields] OR "potentiated"[All Fields] OR "potentiates"[All Fields] OR "potentiating"[All Fields] OR "potentiation"[All Fields] OR "potentiations"[All Fields] OR "potentiative"[All Fields] OR "potentiator"[All Fields] OR "potentiators"[All Fields]) |
| 17 | All combined | 46 | ("apical papilla"[All Fields] OR "pulp"[All Fields] OR "periodontal ligament"[All Fields] OR "dental follicle"[All Fields] OR "dental epithelial"[All Fields] OR "cervical loop"[All Fields] OR "gingival"[All Fields] OR "dental stem cell*"[All Fields]) AND ("sphere"[All Fields] OR (("dental health services"[MeSH Terms] OR ("dental"[All Fields] AND "health"[All Fields] AND "services"[All Fields]) OR "dental health services"[All Fields] OR "dental"[All Fields] OR "dentally"[All Fields] OR "dentals"[All Fields]) AND ("sphere"[All Fields] OR "sphere s"[All Fields] OR "spheres"[All Fields])) OR (("dental health services"[MeSH Terms] OR ("dental"[All Fields] AND "health"[All Fields] AND "services"[All Fields]) OR "dental health services"[All Fields] OR "dental"[All Fields] OR "dentally"[All Fields] OR "dentals"[All Fields]) AND ("sphere"[All Fields] OR "sphere s"[All Fields] OR "spheres"[All Fields]) AND ("formations"[All Fields] OR "metabolism"[MeSH Terms] OR "metabolism"[All Fields] OR "formation"[All Fields]))) AND ("neural"[All Fields] OR "regeneration"[All Fields] OR "potential"[All Fields] OR "neural regeneration"[All Fields] OR "neural potential"[All Fields]) |
